# When Robots Learn: Artificial Intelligence and the Next Human-Centered Era of Neurorehabilitation

**DOI:** 10.3390/brainsci16060656

**Published:** 2026-06-22

**Authors:** Rocco Salvatore Calabrò, David Perpetuini, Irene Giovanna Aprile

**Affiliations:** 1 IRCCS Centro Neurolesi Bonino Pulejo, 98123 Messina, Italy; 2 Department of Engineering and Geology, University G. D’Annunzio of Chieti-Pescara, 65127 Pescara, Italy; david.perpetuini@unich.it; 3 IRCCS Fondazione Don Carlo Gnocchi, 50143 Florence, Italy; iaprile@dongnocchi.it

Neurological disorders remain among the leading causes of long-term disability, care dependency and loss of healthy life worldwide. Current rehabilitation pathways, constrained by stringent regulatory frameworks, remain inadequate, as they focus predominantly, if not almost exclusively, on the period immediately following an acute event. Rehabilitation should instead be personalized and person-centered, addressing the individual’s specific needs while aiming to achieve optimal functional recovery and social reintegration. The growing global demand for rehabilitation services, together with the increasing need for person-centered rehabilitation models, is placing substantial pressure on healthcare systems, thereby underscoring the urgent need to redesign and innovate current rehabilitation paradigms [1,2]. Rehabilitation is now recognized not as an optional post-acute intervention, but as the third pillar of healthcare systems, as a core health strategy across prevention, injury, recovery, continuum of illness, and long-term functioning [2,3]. This perspective is consistent with the Rehabilitation 2030 agenda, which calls for accessible, integrated and person-centered services capable of improving real-world functioning rather than merely extending survival or reducing impairment in isolated clinical settings [4]. Neurorehabilitation therefore faces a decisive challenge: it must become more intensive, measurable, adaptive and continuous, while remaining clinically meaningful, ethically responsible and accessible to patients with heterogeneous neurological profiles, different levels of disability and variable social support. Furthermore, the need to design and implement economically sustainable rehabilitation models should not be underestimated, as failure to address sustainability may ultimately hinder their real-world adoption and long-term integration into healthcare systems. In this context, artificial intelligence (AI), robotics and digital technologies should not be interpreted as parallel innovations added to conventional care, but as converging tools that may help redefine how recovery is assessed, supported, personalized and maintained over time.

The most relevant question is not whether a robot can deliver more repetitions than a therapist, or whether an algorithm can classify more variables than a clinical scale, but whether intelligent, supervised and human-centered systems can help clinicians understand the patient’s changing condition and adapt rehabilitation accordingly. Robotics has traditionally offered important advantages in terms of repetitive practice, task-specific training, standardized assistance, objective measurement and high-dose motor exercise, especially in individuals who require intensive upper-limb or gait rehabilitation.

However, the true turning point emerges when robotic systems are integrated with AI, as we wanted to demonstrate with our Special Issue. In this framework, the rehabilitation robot may evolve from a device that executes pre-programmed movements into a learning system able to estimate performance, modulate assistance, adjust task difficulty, shape feedback, monitor safety and support clinical reasoning through multimodal data. Such a transformation is particularly relevant because neurological recovery is rarely linear. Patients with similar diagnoses may differ substantially in residual motor control, spasticity, fatigue, pain, cognition, motivation, attention, sensory feedback, emotional state and capacity for practice. An intelligent robot supervised by an expert clinician could integrate kinematic, kinetic, electromyographic, wearable-derived, neurophysiological and clinical data to identify trajectories of response and non-response, refine treatment intensity, prevent ineffective dose escalation and support more precise rehabilitation planning. The goal is not the autonomous replacement of the therapist, but the development of assistive intelligence designed to support clinical decision-making and enhance rehabilitation care: a model in which technology extends observation, supports personalization and improves continuity, while the clinician remains responsible for interpretation, goal setting, safety, therapeutic alliance and ethical decision-making.

Recent studies illustrate how this transition is already beginning to take shape. In stroke rehabilitation, the integration of machine learning with robotic rehabilitation has been proposed as a way to support prediction of upper-limb motor recovery, suggesting that data generated during robotic training may have prognostic value when interpreted through appropriate computational models [5]. Comparative work on robotic and non-robotic telerehabilitation for subacute upper-limb disability after stroke further indicates that the clinical value of technology-supported care should not be judged only by impairment-level change, but also by accessibility, autonomy, adherence, quality of life and integration into real-world pathways [6]. Similarly, immersive virtual reality combined with robot-assisted gait training has shown feasibility and acceptability in individuals with neurological diseases, emphasizing that ecological feedback, motivation and user experience are not secondary elements, but central mediators of engagement and sustained practice [7].

Beyond motor output, AI also expands what rehabilitation systems may learn from the brain and from complex behavioral states. EEG-based machine learning has been used for multidimensional assessment, severity estimation and cognitive decline prediction in depression, providing an example of how neurophysiological data can be translated into interpretable support for clinical stratification (Contribution 1). Multimodal MRI–EEG digital twin approaches have also been proposed as patient-specific models for real-time brain health intelligence, highlighting the potential of continuously updated, explainable systems that integrate structural, functional and electrophysiological information (Contribution 2). These important observations derived from explanatory studies are highly relevant to the future of neurorehabilitation, as they demonstrate that a deeper understanding of the relationship between recovery and multimodal biomarkers, beyond biomarkers exclusively related to movement, represents a fundamental prerequisite for the development of innovative adaptive robotic and digital platforms. The same principle applies to virtual reality, digital therapeutics and cognitive rehabilitation. Immersive virtual reality assessment in multiple sclerosis has shown the importance of patient-reported experience, tolerability and perceived relevance, reminding us that digital neurorehabilitation must be evaluated not only through performance metrics, but also through the lived experience of users [8]. A case series on digital neurohabilitation for attention and memory in pediatric obesity should be interpreted cautiously, yet it illustrates the growing interest in app-based and platform-based cognitive interventions that may complement conventional rehabilitation when appropriately designed, monitored and validated (Contribution 3). These examples are relevant even for robotics because sustained motor recovery is influenced by cognition, attention, motivation and fatigue; a robot that adapts only to joint trajectories but ignores cognitive load or perceived effort remains only partially intelligent. Other recent work further supports the need to embed robotics within multimodal rehabilitation pathways rather than presenting it as a standalone solution. A case report combining robot-assisted gait training with conventional physical and occupational therapy in acute dermatomyositis cautiously illustrates how robotic gait technologies may be incorporated into broader functional recovery programs beyond the most traditional neurological populations (Contribution 4). Robotic verticalization training in patients with minimally conscious state has linked intervention to neurophysiological markers and the possibility of neuroplasticity-informed rehabilitation in disorders of consciousness, a field in which objective biomarkers and careful interpretation are particularly important (Contribution 5).

In spinal cord injury, an advanced multidisciplinary rehabilitation pathway combining technologies, clinical expertise and continuity of care has shown encouraging results, reinforcing the idea that technology becomes meaningful when it is organized around patient-centered goals and embedded in a coherent therapeutic environment (Contribution 6). AI may also help rehabilitation address symptoms that are difficult to capture with conventional scales. Fatigue is a paradigmatic example because it is multidimensional, fluctuating and shaped by biological, psychological, cognitive and behavioral factors.

Work on the challenges and promises of AI for fatigue has emphasized that computational models may help integrate heterogeneous data, but also that such models can generate false certainty if they are not clinically interpretable, externally validated and grounded in meaningful outcomes (Contribution 7). This warning applies directly to AI-driven robotics. More data do not automatically produce better rehabilitation. Sensors, algorithms and digital biomarkers must be accurate, transparent, secure, equitable and actionable within clinical workflows.

The next bottleneck is therefore not only technological invention, but clinical implementation. Algorithms require external validation across diverse populations; robotic protocols require pragmatic evaluation against meaningful comparators; digital biomarkers must be linked to function, participation and quality of life; and hybrid models must demonstrate safety, usability, cost-effectiveness and equity. Explainability should be considered a clinical requirement rather than a technical luxury, especially when model outputs influence treatment intensity, prognosis, risk monitoring or discharge planning. Data governance, cybersecurity, privacy, algorithmic bias and the digital divide must also be addressed from the beginning, because technology is never neutral when it is trained on narrow datasets, deployed only in well-resourced centers or designed without patients, caregivers and clinicians.

The future of neurorehabilitation will not be defined by machines replacing professionals. It will be defined by intelligent, accountable and human-centered systems that allow clinicians, patients and caregivers to deliver rehabilitation that is more adaptive, measurable, intensive and meaningful. When robots learn, they should learn under clinical supervision, in the service of human goals, and with a clear commitment to safety, equity and real-world functioning. This is the most promising and most demanding frontier of AI-driven neurorehabilitation.

## Data Availability

No new data were created or analyzed in this article.
